# Innate and Adaptive Immunity-Related Markers as Predictors of the Short-Term Progression of Subclinical Atherosclerosis in Middle-Aged Patients

**DOI:** 10.3390/ijms241512205

**Published:** 2023-07-30

**Authors:** Vadim Genkel, Ilya Dolgushin, Albina Savochkina, Karina Nikushkina, Irina Baturina, Anna Minasova, Veronika Sumerkina, Lubov Pykhova, Semen Kupriyanov, Alla Kuznetsova, Igor Shaposhnik

**Affiliations:** Federal State Budgetary Educational Institution of Higher Education “South-Ural State Medical University” of the Ministry of Healthcare of the Russian Federation, 454092 Chelyabinsk, Russia; dol-ii@mail.ru (I.D.); alina7423@mail.ru (A.S.); knikushkina81@gmail.com (K.N.); irisha_baturina@mail.ru (I.B.); pandora_anna@mail.ru (A.M.); veronika.sumerkina@mail.ru (V.S.); lyubov_pykhova@mail.ru (L.P.); pfft@mail.ru (S.K.); kuzja321@mail.ru (A.K.); shaposhnik@yandex.ru (I.S.)

**Keywords:** atherosclerosis, inflammation, intermediate monocytes, immunosenescence, Toll-like receptors

## Abstract

Assessment of inflammation is a promising approach to monitoring the progression of asymptomatic atherosclerosis. The aim of the present study was to investigate the predictive value of innate and adaptive immunity-related markers, in relation to the short-term progression of subclinical atherosclerosis. The study included 183 patients aged 40–64 years who underwent duplex scanning of the carotid and lower limb arteries at two visits with an interval of 12–24 months between examinations. Phenotyping of circulating lymphocytes and monocytes subpopulations were performed through flow cytometry. An increase in the number of circulating TLR4-positive intermediate monocytes (>447.0–467.0 cells/μL) was an independent predictor of the short-term progression of lower limb artery atherosclerosis (*p* < 0.0001) and polyvascular atherosclerosis (*p* = 0.003). The assessment of TLR4-positive monocytes significantly improved the prognostic model for the progression of lower limb arterial atherosclerosis (C-index 0.728 (0.642–0.815) versus 0.637 (0.539–0.735); *p* = 0.038). An increase in the number of circulating TLR4-positive intermediate monocytes was an independent predictor of the short-term progression of lower limb artery and polyvascular atherosclerosis. Their inclusion into models containing conventional risk factors significantly improved their prognostic effectiveness regarding lower limb artery atherosclerosis progression.

## 1. Introduction

The results of studies using dynamic coronary angiography before and after acute myocardial infarction made it possible to establish the fact that the development of myocardial infarction is preceded by a period in which there is a rapid progression of atherosclerosis with the atherosclerotic plaque growth and an increase in the degree of vessel stenosis [1]. These findings have led to relevant research focused on the significance of subclinical atherosclerosis progression as a predictor of long-term major adverse cardiovascular events (MACE) [2]. Moreover, these data serve as a strong argument in favor of the potential effectiveness of image-guided cardiovascular prevention [3]. In numerous clinical studies it has been shown that the short-term progression of subclinical carotid atherosclerosis may be directly related to an increase in the relative risk (RR) of MACE in the long term. One of the first and largest relevant research studies was initiated in Taiwan and carried out from 1994 to 1995 (the Chin-Shan Community Cardiovascular Cohort Study (CCCC) [4]. According to the results of univariate Cox regression analysis, the appearance of a new carotid plaque during 5 years follow-up was directly associated with an increase in the RR of MACE after 13 years: combined endpoint (stroke and coronary events) increase by 2.26 times (95% CI 1.51–3.38); stroke by 2.51 times (95% CI 1.48–4.28); and coronary events (coronary death, nonfatal myocardial infarction and coronary revascularization) by 1.95 times (95% CI 1.07–3.55). In a study by E.Y. Lim et al. reported that in patients after strokes or transient ischemic attacks (TIA), carotid atherosclerosis progression was shown to be over 35.0 ± 22.6 months of follow-up and independently associated with a 3.8-fold increase in the RR of recurrent stroke (95% CI 1.1–13.1; *p* = 0.034) [5].

Chronic systemic non-resolving sterile inflammation is the most important mechanism associated with the development and progression of atherosclerosis [6]. The main trigger of inflammation in atherosclerosis is apoB-containing lipoproteins, whose accumulation in the subendothelial space of the vascular wall initiates an inflammatory response involving innate and adaptive immune cells [7]. The accumulation of apoB-containing lipoproteins in the vascular wall with their subsequent modification, along with the recruitment of immune cells into the vascular wall and their activation, are probably the main drivers behind the progression of atherosclerosis [8,9]. Currently, it is known that in atherosclerosis, inflammation occurs both at the local and systemic levels [10,11]. New technologies and approaches, such as the multi-omics approach and single cell sequencing, have made significant progress in understanding the immune mechanisms of atherosclerosis and have broadened the understanding of the involvement of multiple cells of innate and adaptive immunity in atherogenesis [12]. The rapid evolution of single-cell transcriptomics and proteomics techniques has provided insight into the cellular landscape of atherosclerosis and identified novel subpopulations of immune cells associated with the progression of atherosclerosis in humans [13,14]. In clinical studies and, within the perspective future clinical practice, the study of circulating immune cells and serum biomarkers is the most easily feasible and promising direction for monitoring the progression of atherosclerosis and assessing the risk of future cardiovascular events [15,16]. The development and implementation of multiparametric panels in clinical practice is an emerging pathway to precision cardiovascular prevention and therapy [15]. The aim of the present study was to investigate the predictive value of innate and adaptive immunity-related markers, in relation to the short-term progression of subclinical atherosclerosis.

## 2. Results

The study included 183 patients, the clinical characteristics of which are shown in Table 1. 

According to the assessment of initial cardiovascular risk (CVR) before arterial duplex scanning, 48 (26.2.%) patients were classified as having a low CVR, 76 (41.5.%) as having a moderate CVR, 28 (15.3.%) as possessing a high CVR and 31 (16.9.%) as having a very high CVR [17]. It should be noted that 44.8.% of patients had polyvascular atherosclerosis, and 14.8.% had generalized polyvascular atherosclerosis (≥4 vascular territories affected) [18]. The results of serum markers of inflammation are summarized in Table 2.

Table 3 shows the results of flow cytometry.

### 2.1. Short-Term Progression of Subclinical Atherosclerosis and Its Clinical Predictors

The median time interval between the first and second visit with peripheral arterial DS was 15.5 (12.1; 23.5) months. Progression of carotid atherosclerosis was registered in 83 (45.3%) patients, lower limb artery atherosclerosis—in 66 (36.0%) patients, and polyvascular atherosclerosis in 41 (22.4%) patients.

According to the Cox univariate regression analysis, the male sex was associated with an increase of 1.74 times in the RR of carotid atherosclerosis progression (95% CI 1.12–2.71; *p* = 0.013), an increase of 1.81 times in the RR of lower limb arterial atherosclerosis progression (95% CI 1.10–2.97; *p* = 0.020), and an increase of 3.05 times with regard to polyvascular atherosclerosis progression (95% CI 1.54–6.03; *p* = 0.001). In addition, smoking (RR 1.95 (95% CI 1.18–3.22; *p* = 0.009) and young age (0.96 (0.93–0.99; *p* = 0.011)) were also shown to be associated with the increased RR of carotid atherosclerosis progression. There were no statistically significant associations between other cardiovascular risk factors and the progression of atherosclerosis. The model which included the male sex, age, and smoking showed a rather low level of prognostic effectiveness in relation to the progression of carotid atherosclerosis (C-index 0.598 (0.525–0.670); *p* = 0.003), lower limb artery atherosclerosis (C-index 0.637 (0.539–0.735); *p* = 0.20) and polyvascular atherosclerosis (C-index 0.651 (0.542–0.759); *p* = 0.10).

### 2.2. Serum Markers of Inflammation as Predictors of the Short-Term Progression of Atherosclerosis

**Figure 1:**
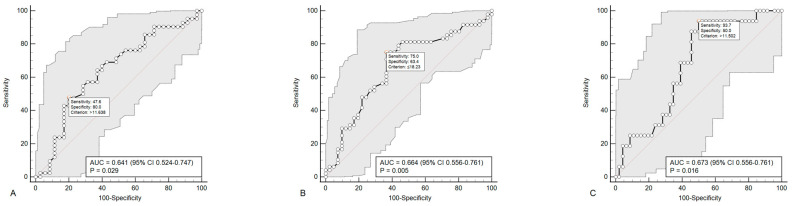
ROC curves demonstrating the diagnostic efficacy of pentraxin 3 (**A**) and IFNγ (**B**) on carotid atherosclerosis progression, TGFβ—on polyvascular atherosclerosis progression (**C**).

In subgroup comparisons, it was found that patients with progression of carotid atherosclerosis had a higher serum concentration of pentraxin 3 (11.4 pg/mL (6.70–13.9) versus 6.82 pg/mL (2.54–11.5) (*p* = 0.034)) and lower IFNγ (14.9 pg/mL (10.4–18.7) versus 20.8 pg/mL (14.8–25.3) (*p* = 0.008)). Patients with polyvascular atherosclerosis progression had high serum TGFβ concentrations (26.2 pg/mL (17.1–43.7) versus 12.2 pg/mL (6.01–36.1) (*p* = 0.040)). In order to assess the diagnostic efficiency of pentraxin 3, IFNy, and TGFβ on the progression of subclinical atherosclerosis, an ROC analysis was performed, the results of which are shown in Figure 1.

According to the results of univariate and multivariate Cox regression analysis, an increase in pentraxin 3 above certain cut-off values and a decrease in IFNγ below certain cut-off values did not significantly increase the RR of carotid atherosclerosis progression. However, an increase in TGFβ > 11.5 pg/mL was associated with an increase of 7.60 times in the RR of polyvascular atherosclerosis progression (95% CI 1.001–57.7; *p* = 0.0499) according to the univariate analysis. In the model adjusted for male sex, or male sex, age, and smoking, the relationship between TGFβ concentration and the progression of polyvascular atherosclerosis became statistically insignificant (*p* = 0.0750). On the other hand, adding TGFβ as a quantitative variable to the model that included male sex, age, and smoking resulted in an increase in its predictive effectiveness (C-index 0.720 (0.561–0.878); *p* = 0.04). Although the model became statistically significant, the increase in the C-index, when compared with the baseline model, was not significant (*p* = 0.266).

### 2.3. Circulating Innate and Adaptive Immune Cells as Predictors of the Short-Term Progression of Atherosclerosis

**Figure 2:**
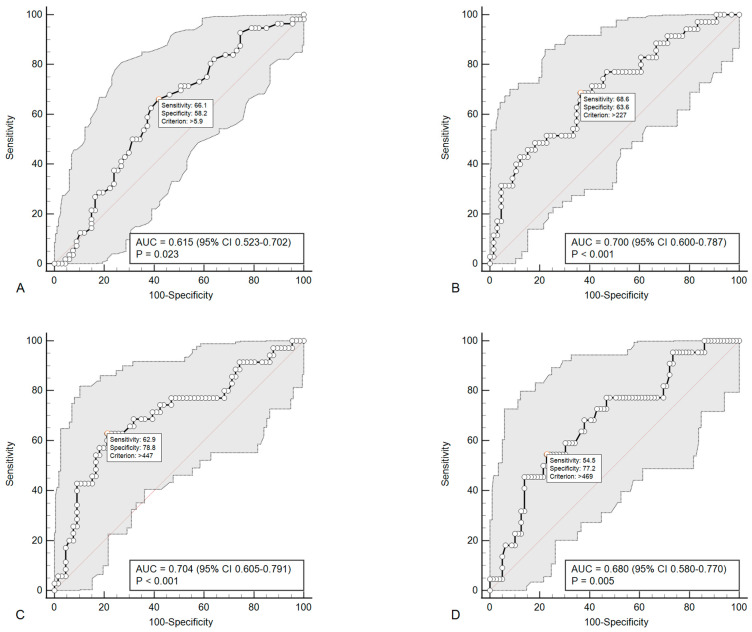
ROC curves demonstrating the diagnostic efficacy of T-regulatory lymphocytes in relation to carotid atherosclerosis progression (**A**), TLR4-positive classical monocytes in relation to lower limb arterial atherosclerosis progression (**B**), TLR4-positive intermediate monocytes in relation to lower limb artery atherosclerosis (**C**), and polyvascular (**D**) atherosclerosis progression.

A subgroup comparison showed that patients with progression of carotid atherosclerosis had higher relative numbers of T-regulatory (CD4^+^CD4^+^CD25^+^CD127^−^) cells (6.55% (5.25–8.30) versus 5.80% (5.20–6.10), *p* = 0.0278). Patients with progression of lower limb artery atherosclerosis characterized by greater relative (67.1% (54.9–77.5) vs. 41.1% (29.3 vs. 68.2); *p* = 0.047) and absolute (328.0 cells/μL (179.5–475.2) vs. 167.5 cells/μL (128.0–221.3); *p* = 0. 001) number of TLR4-positive classical monocytes (CD14^++^CD16^−^TLR4^+^), and higher absolute (475.0 cells/μL (376.7–551.2) vs. 363.0 cells/μL (331.6–409.0); *p* = 0.0008) number of TLR4-positive intermediate monocytes (CD14^++^CD16^+^TLR4^+^). An increase in the absolute number of TLR4-positive intermediate monocytes (CD14^++^CD16^+^TLR4^+^) was also found in patients with polyvascular atherosclerosis progression—479.0 cells/μL (388.0–543.0) versus 761.0 cells/μL (273.2–466.5), *p* = 0.0099. To assess the diagnostic effectiveness of these cell subpopulations in relation to the progression of atherosclerosis, an ROC analysis was carried out (see Figure 2).

According to the Cox regression analysis, only an increase in the number of circulating TLR4-positive intermediate monocytes was associated with an increase in the RR of atherosclerosis progression. It was found that an increase in their absolute number > 447.0 cells/µL was associated with an increase by 4.28 times in the RR of lower limb artery atherosclerosis progression (95% CI 2.09–8.79; *p* < 0.0001) after adjustments for sex, age and smoking. An increase in the number of circulating TLR4-positive intermediate monocytes > 467.0 cells/µL was also associated with an increase of 3.95 times in the RR of polyvascular atherosclerosis progression (95% CI 1.62–9.66; *p* = 0.003) after adjusting for sex, age, and smoking. Adding the number of TLR4-positive intermediate monocytes as a quantitative variable in the model used for predicting the progression of lower limb atherosclerosis and polyvascular atherosclerosis, which includes male sex, age, and smoking, led to an increase in their prognostic effectiveness (C-index 0.728 (0.642–0.815); *p* = 0.0006 and 0.725 (0.613–0.838); *p* = 0.001, respectively). Although both models became statistically significant, only for the model predicting progression of lower limb artery atherosclerosis an increase in the C-index was significant (*p* = 0.038), but not for the model predicting progression of multivessel atherosclerosis (*p* = 0.112).

### 2.4. Relationships between TGFβ, T-Regulatory Lymphocytes, and TLR4-Positive Intermediate Monocytes with Other Immunity-Related Markers and Indicators of Plaque Burden

We analyzed the relationships of TGFβ and TLR4-positive intermediate monocytes (as indicators that showed independent prognostic value in relation to the progression of atherosclerosis) as well as T-regulatory lymphocytes, with other immunity-related markers and indicators of plaque burden. Thus, the serum concentration of TGFβ was inversely correlated with the absolute number of CD3^+^CD25^+^ (r = −0.493; *p* < 0.0001), CD8^+^CD25^+^ (r = −0.377; *p* = 0.008), CD8^+^HLA-DR^+^ (r = −0.418; *p* = 0.003) and directly correlated with cTPA (r = 0.308; *p* = 0.033). An increase in the absolute number of T-regulatory lymphocytes was associated with an increase in CD3^+^ (r = 0.472; *p* < 0.0001), CD3^+^CD25^+^ (r = 0.203; *p* = 0.029), CD3^+^CD8^+^ (r = 0.313; *p* = 0.001) and maximal stenosis of lower limb arteries (r = 0.302; *p* = 0.007). The absolute number of circulating CD14^++^CD16^+^TLR4^+^ had a direct correlation with the absolute number of CD3^+^ (r = 0.377; *p* < 0.0001), CD3^+^CD8^+^ (r = 0.232; *p* = 0.021), and maximal stenosis of lower limb arteries (r = 0.387; *p* = 0.002) and total plaque number (r = 0.219; *p* = 0.030). In addition, total plaque number directly correlated with the concentration of pentraxin 3 (r = 0.272; *p* = 0.019).

**Figure 3:**
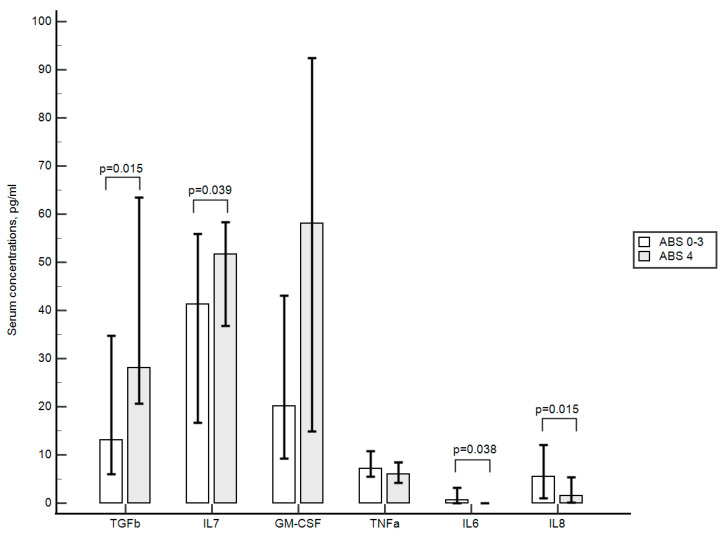
TGFβ, IL7, GM-CSF, TNFα, IL6, IL8 concentration in relation to the presence of generalized polyvascular atherosclerosis. Pairwise comparisons were performed using the Mann–Whitney test.

When analyzing the intergroup differences, it was found that patients with generalized polyvascular atherosclerosis had significantly higher concentrations of TGFβ and IL7 and lower concentrations of IL6 and IL8, while differences in the content of GM-CSF (*p* = 0.071), TNFα (*p* = 0.059), and other cytokines did not reach levels of statistical significance (see Figure 3).

**Figure 4:**
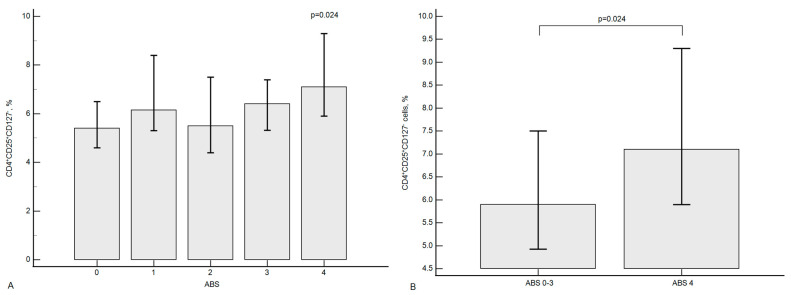
T-regulatory lymphocyte levels as a function of atherosclerosis extent (**A**) and the presence of generalized polyvascular atherosclerosis (**B**). Comparisons between the five groups (**A**) were made using the Kruskal–Wallis test, followed by post hoc Dunn’s test with the Bonferroni significance level correction. Pairwise comparisons (**B**) were performed using the Mann–Whitney test.

It should be noted that the number of T-regulatory lymphocytes also significantly changed depending on the extent of subclinical atherosclerosis (see Figure 4).

Thus, it was found that as the number of vascular territories involved increases, an initial increase in T-regulatory lymphocytes is observed, followed by a decrease and then an increase during the development of generalized atherosclerosis (pairwise comparison in five subgroups (Figure 4A) using the Dunn’s test with the Bonferroni significance level correction (*p* < 0.005) did not reveal significant differences). 

## 3. Discussion

The tools being currently used for predicting cardiovascular events demonstrate limited accuracy at the individual-patient level [19]. Assessment of inflammation, which integrates the negative effects of traditional risk factors, which are upstream triggers of vascular inflammation, is a promising tool for personalizing cardiovascular prevention [19,20]. From a clinical point of view, the measurement of one or more inflammation markers (for example, hsCRP) is the preferred option for assessing residual inflammatory risk. However, it is clearly obvious that this approach has extremely limited additional value since it does not allow adequate assessment of the complex dynamics of the immune system and the activation of certain critical inflammatory pathways during atherosclerosis progression and the inflammatory response evolution [21]. 

The main findings of the study being presented are: (1) an increase in TGFβ > 11.5 pg/mL was associated with an increase of 7.60 times in the RR of polyvascular atherosclerosis progression (95% CI 1.001–57.7; *p* = 0.0499) with the inclusion of this indicator in the model containing traditional risk factors resulting in its statistical significance; (2) an increase in the number of circulating TLR4-positive intermediate monocytes was an independent predictor of the short-term progression of lower limb artery atherosclerosis and polyvascular atherosclerosis, and their inclusion in models containing traditional risk factors made it possible to significantly improve their prognostic effectiveness; (3) patients with a short-term progression of subclinical atherosclerosis were characterized by a higher serum concentration of pentraxin 3 and a lower concentration of IFNγ, and a higher relative number of T-regulatory cells. However, these parameters were not connected to an increase in the RR of atherosclerosis progression; (4) an increase in TGFβ and the number of T-regulatory lymphocytes was shown to be associated with an increase in the burden of systemic atherosclerosis and may probably represent the compensatory activation of immunosuppressive immune networks.

The results of numerous studies have shown that in atherosclerosis, there is an increase in the number of circulating CD16^+^ monocytes, i.e., intermediate and non-classical monocytes [22]. Currently, it is intermediate monocytes that are considered ‘inflammatory monocytes’ and the expression of TLRs, including TLR4, is highest on them compared to other monocyte subpopulations [23]. Intermediate monocytes have the greatest ability to produce reactive oxygen species, proinflammatory cytokines, and activate CD4^+^ cell proliferation [23,24]. In studies by M. Wildgruber et al. demonstrated a significant increase in the number of circulating intermediate monocytes in lower limb peripheral artery disease, as well as the predictive value of this increase in relation to restenosis 12 months after balloon angioplasty of the lower limb arteries [22,25]. A group of researchers led by S.N. Pokrovsky and M.V. Ezhov found that an increase in the number of both intermediate monocytes and non-classical monocytes in patients with elevated lipoprotein(a) significantly increased the odds ratio of the presence of three-vessel coronary atherosclerosis [26]. In several clinical studies, the prognostic significance of an increase in intermediate monocytes in relation to the adverse cardiovascular events in patients on dialysis and patients undergoing coronary angiography has been shown [24]. At the same time, according to the available data, for the first time we have established the prognostic significance of TLR4-positive intermediate monocytes in relation to the short-term progression of polyvascular atherosclerosis.

In the current study, an increase in the number of T-regulatory lymphocytes and TGFβ was associated with an increase in the burden of systemic atherosclerosis, and an increase in TGFβ was also associated with an increase of 7.60 times in the RR of polyvascular atherosclerosis progression. In addition, an increase in the serum concentration of TGFβ was associated with a decrease in the number of activated T-cells and activated T-cytotoxic lymphocytes. Further, it was found that patients with generalized atherosclerosis, in addition to a statistically significant increase in T-regulatory lymphocytes, TGFβ and IL7, also had a significant decrease in concentrations of IL6 and IL8. In our opinion, these findings can represent the activation of the anti-inflammatory network of immunity, accompanied on the other hand by an increase in the number of “inflammatory” monocytes and pentraxin 3. It is important to note that pro-inflammatory and anti-inflammatory markers were closely related and associated with the progression of subclinical atherosclerosis. It is known that an increase in IL7 contributes to the survival of circulating T-regulatory lymphocytes and an increase in their number [27,28]. T-regulatory lymphocytes, in turn, are the most important source of TGFβ, which implements its anti-inflammatory properties, including inhibiting the production of effector pro-inflammatory cytokines, such as IFNγ, IL6, and others [29,30,31,32]. Being different aspects of the same process, chronic systemic inflammation, increases in both pro-inflammatory and anti-inflammatory markers can represent the burden of systemic atherosclerosis and be associated with its progression. Moreover, in our opinion, the established findings can be adequately described within the framework of the concepts of “Immunosenescence” and “Inflammaging” [33,34]. 

It is assumed that the “IL7-IL7R” axis probably plays an important role in the processes of immunosenescence and that its assessment can be used as a diagnostic tool for this condition [35]. According to V. Nguyen et al., despite the fact that IL7 is a necessary factor for “lymphocyte homeostasis” and its effects on normal aging, a persistent increase in IL7 above the physiological level can disrupt this delicate balance [35]. This position is also supported by the fact that for the implementation of the homeostatic functions of IL7, intermittent signaling is required and not a persistent increase [36,37]. Changes in the landscape of circulating CD4^+^ cells are also a characteristic feature of chronic systemic inflammation and immunosenescence [38,39]. Thus, in experimental studies, a significant pronounced increase in cytotoxic T-lymphocytes, exhausted T-cells and T-regulatory lymphocytes was found [39]. At the same time, it is not completely clear what the source of T-regulatory lymphocytes is under conditions of thymus involution, which is one of the key events of immunosenescence. Taking into account the pattern of gene expression, exhausted T-lymphocytes are a possible source of T-regulatory lymphocytes [39]. The functional status of such T-regulatory lymphocytes is characterized by greater immunosuppressive activity [40]. Clinical studies have also confirmed the accumulation of T-regulatory lymphocytes in connection to chronological aging and the association of T-regulatory cells with immunosenescence [41,42]. The expansion of T-regulatory lymphocytes and myeloid derived suppressor cells (MDSC), promotes an increase in TGFβ which is a key regulator of physiological and pathological aging, fibrosis and atherogenesis [43].

The results of clinical studies have demonstrated that an increase in factors conditionally related to the “anti-inflammatory” cluster correlates with a more pronounced violation of physiological functions and a worsened prognosis [44,45]. However, what is important is most likely not the negative impact of these factors per se, but their ability to reflect the adaptive response of the anti-inflammatory network of the immunity and its insufficient effectiveness in suppressing inflammation below the threshold associated with ongoing cell and tissue damage [44]. On the other hand, activation of the immunosuppressive network stimulates further immunosenescence. Despite the fact that there is currently no consensus on whether immunosenescence is a cause or in fact a consequence of inflammaging, most authors consider chronic inflammation and inflammaging to be of primary importance, since signs of immunosenescence can be detected in various inflammatory diseases regardless of chronological age [45]. It is possible that the development of protocols which are able to assess both chronic inflammation and immunosenescence will have additional prognostic value with regard to patients with atherosclerosis. 

The presented study has a number of important limitations: (1) a mixed sample of patients aged 40–64 years, including both patients with low CVR and patients with atherosclerotic CVD; and (2) a mixed sample of patients with regard to the therapy received. This requires a replication of the results with other samplings of patients.

## 4. Materials and Methods

The study included patients aged 40–64 years who underwent duplex scanning (DS) of the carotid and lower limb arteries at two visits with an interval of 12–24 months between examinations. After being enrolled into the study, all patients signed an informed consent form. The study protocol was approved by the Ethics Committee of South-Ural State Medical University (Protocol No. 10 of 27.10.2018). The study exclusion criteria included the presence of the following clinical conditions: myocardial infarction and/or stroke in the previous 28 days; severe dysfunction of the liver and kidneys (decreased estimated glomerular filtration rate (eGFR) less than 30 mL/min/1.73 m^2^); malignant neoplasms; diagnosed chronic inflammatory diseases (CID) and/or the presence of acute inflammatory or infectious diseases during the previous 28 days.

### 4.1. Duplex Scanning

All patients underwent duplex scanning of the carotid and the lower limb arteries. The study was carried out in B-mode, color mapping mode, and pulse Doppler mode. The following vessels were examined from both sides in longitudinal and transverse sections throughout their length: the common carotid arteries (CCA) with CCA bifurcation, the internal carotid arteries (ICA), the external carotid arteries (ECA), common femoral arteries (CFA), superficial femoral arteries (SFA), the popliteal arteries (PA), the tibioperoneal trunk, the anterior tibial arteries, and the posterior tibial arteries. 

Atherosclerotic plaque was defined as focal thickening of the IMT more than 1.5 mm or 0.5 mm larger than the surrounding IMT or 50% larger than the IMT of the adjacent areas [46]. The percentage of stenosis was measured planimetrically in B-mode by diameter in the cross section of the vessel. Stenosis percentages were determined according to the European Carotid Surgery Trial (ECST) method. In the case of detection of plaque, stenosing the lumen of the vessels was carried out and the maximum percentage of stenosis in a particular patient was determined. The examination was carried out with a linear probe with a frequency of 10 MHz using Canon Aplio 400 (Tokyo, Japan) digital ultrasonic multifunctional diagnostic scanner.

The following plaque burden indicators were determined: carotid total plaque area (cTPA), carotid total plaque thickness (carotid plaque score, cPS), number of carotid and femoral bifurcations with plaque (atherosclerosis burden score, ABS) [47]. The technique for measuring these indicators has been described previously [48,49]. The number of plaques in carotid and in the lower limb arteries and the total number of plaques were also determined.

In order to assess the progression of carotid atherosclerosis through the dynamics of changes in the cTPA and cPS and minimize the influence of uncertainty in measurement, the measurement error was calculated for these indicators (measurement of cTPA and cPS was carried out in 20 patients with an interval of 24 and 48 h). The measurement error (σ) was equal to three standard deviations of the difference between two measurements taken 24 and 48 h apart [50,51]. The measurement error of the cTPA and cPS was 0.053 cm^2^ and 0.16 mm, respectively. Progression was considered to be an increase in the cTPA and/or a cPS > 2σ, i.e., by more than 0.106 cm^2^ and 0.32 mm, respectively [50]. It is known that 1.96 σ includes 95% of errors, which makes it possible to determine, with a high degree of probability, the true increase in the plaque burden [51]. Thus, the relevant criteria for the progression of subclinical atherosclerosis were: (1) the appearance of new atherosclerotic plaque in the carotid arteries and/or the arteries of the lower limbs; (2) an increase in the degree of stenosing in previously existing stenosis ≥ 10%; and (3) an increase in the cTPA by more than 0.106 cm^2^ and/or an increase in the cPS more than 0.32 mm. To classify a patient as a progressor, a ≥ 1 criterion was required.

### 4.2. Laboratory Examinations

All patients underwent fasting blood count tests with an automatic analyser (Medonic M16, Boule Medical A.B., Spånga, Sweden), for which their venous blood was collected into tubes containing the K2 EDTA.

The following biochemical laboratory blood parameters were obtained after fasting for at least 8 h: TC, LDL-C, HDL-C, TG, glycated haemoglobin, and creatinine with subsequent eGFR calculation according to the CKD-EPI formula (BioChem Analette, North Attleboro, MA, USA).

The concentration of high-sensitivity C-reactive protein (hsCRP) in blood serum was measured using an enzyme-linked immunosorbent assay (VECTOR-BEST, Novosibirsk, Russia). The concentration of pentraxin 3 in blood serum was measured using an enzyme-linked immunosorbent assay (Human Pentraxin 3/PTX3 ELISA Kit (ab214570, Abcam, Cambridge, UK). The concentration of transforming growth factor beta (TGFβ) in blood serum was measured using an enzyme-linked immunosorbent assay (Human TGF-beta 1 ELISA Kit (ab100647, Abcam, Cambridge, UK). The ELISA was performed on automatic enzyme-linked immunosorbent assay Personal LAB (Adaltis, Rome, Italy). The concentration of granulocyte-macrophage colony-stimulating factor (GM-CSF), interferon γ (IFNγ), interleukin-1β (IL1β), interleukin-2 (IL-2), interleukin-4 (IL4), interleukin-5 (IL5), interleukin-6 (IL6), interleukin-7 (interleukin-7), interleukin-8 (IL8), interleukin-10, (IL10), interleukin-12 (IL12p70), interleukin-13 (IL13), tumor necrosis factor-alpha (TNFα) was measured using were performed by multiplex analysis using xMAP technology (Luminex, Austin, TX, USA) with MILLIPLEX MAP Human High Sensitivity T Cell Bead-Based Multiplex Assays (HSTCMAG28SPMX13, Merck-Millipore, Burlington, VT, USA).

Phenotyping of circulating lymphocytes and monocytes subpopulations were performed through flow cytometry (Navios 6/2, Beckman Coulter, Brea, CA, USA). Blood was collected after fasting for at least 8 hours into K2 EDTA tubes. For the phenotyping of lymphocytes and monocytes subpopulations, conjugated monoclonal antibodies were used: CD3, PE-eFluor 610 (eBioscience, San Diego, CA, USA); CD19, PE (eBioscience, San Diego, CA, USA); CD56, PE (eBioscience, San Diego, CA, USA); CD4, APC (eBioscience, San Diego, CA, USA); CD8, PE-Cy5.5 (Invitrogen, Waltham, USA); CD25, PE-Cy7, eBioscience, San Diego, CA, USA); HLA-DR, APC (eBioscience, San Diego, CA, USA); CD11b, FITC (eBioscience, San Diego, CA, USA); CD127, FITC, eBioscience, San Diego, CA, USA); CD14, PerCP-Cy5.5 (eBioscience, San Diego, CA, USA); CD16, PE-Cy7 (eBioscience, San Diego, CA, USA); CD282, Alexa Flour 647 (BioLegend, San Diego, CA, USA); CD284, PE (BioLegend, San Diego, CA, USA); CD36, FITC (BD Biosciences, San Diego, CA, USA). Phenotyping of lymphocytes and monocytes was performed in whole blood with detection of at least 30,000 events. Examples of gating strategies are presented in the supplementary data (see Figures S1–S5).

### 4.3. Statistical Analysis

The data that were obtained were analyzed using the statistical data analysis package MedCalc (ver. 20.019, MedCalc Software Ltd., Osten, Belgium) and IBM SPSS Statistics (v. 18, SPSS Inc., Chicago, IL, USA). Qualitative variables were described by absolute and relative frequencies (percentages). Quantitative variables were described by the median (Me) indicating the interquartile interval [25th percentile and 75th percentile]. Spearman’s correlation analysis was used to determine the relationship between the indicators. Any significant differences between more than two groups were assessed using the Kruskal–Wallis test followed by a pairwise comparison using Dunn’s test. The critical level of significance of differences was assessed by Bonferroni adjustment (*p* = 0.05/number of paired comparisons). Mann–Whitney test was used to compare quantitative measures between the two groups. Differences were considered statistically significant at a critical significance level of 0.05.

In order to establish the threshold values of the studied parameters, receiver operating characteristics (ROC) analysis was performed in order to obtain the determination of sensitivity and specificity. The calculations of the area under the characteristic curve (AUC) with a 95% confidence interval (CI) and were also carried out. To predict the relative risk of an event occurring and to assess the influence of independent variables on this risk, Cox regression analysis was used with the determination of the Harrell C-index to assess the predictive efficiency of the models. The Harrell C-index was calculated and compared for the different models using the statistical package “Survival” for the R programing language.

## 5. Conclusions

In patients aged 40 to 64 years of age, short-term progression of subclinical polyvascular atherosclerosis was observed in 22.4% of patients. An increase in the number of circulating TLR4-positive intermediate monocytes was an independent predictor of the short-term progression of lower limb artery and polyvascular atherosclerosis. Their inclusion into models containing conventional risk factors significantly improved their prognostic effectiveness regarding lower limb artery atherosclerosis progression.

## Figures and Tables

**Table 1:** Clinical characteristics of patients.

Characteristics	Patients (n = 183)
Male, n (%)/Female, n (%)	82 (44.8)/101 (55.2)
Age, years, Me (IQR)	51.0 (45.0; 57.0)
BMI, kg/m^2^, Me (IQR)	26.8 (23.4; 30.1)
Obesity, n (%)	45 (24.7)
Abdominal obesity, n (%)	96 (52.7)
Smoking, n (%)	32 (17.5)
HTN, n (%)	92 (50.3)
T2DM, n (%)	15 (8.20)
CAD, n (%)	31 (16.9)
β-blockers, n (%)	37 (20.2)
RAAS-inhibitors, n (%)	63 (34.4)
Diuretics, n (%)	17 (9.30)
Statins, n (%)	51 (27.9)
Antiplatelets, n (%)	39 (21.3)
TC, mmol/L, Me (IQR)	5.44 (4.65; 6.31)
LDL-c, mmol/L, Me (IQR)	3.46 (2.69; 4.20)
HDL-c, mmol/L, Me (IQR)	1.38 (1.16; 1.59)
Non-HDL-c, mmol/L, Me (IQR)	3.98 (3.33; 4.91)
Triglycerides, mmol/L, Me (IQR)	1.10 (0.83; 1.50)
Glucose, mmol/L, Me (IQR)	5.36 (4.97; 5.90)
HbA1c, %, Me (IQR)	5.60 (5.20; 6.05)
Creatinine, μmol/L, Me (IQR)	94.0 (78.0; 107.7)
eGFR, ml/min/1.73 m^2^, Me (IQR)	71.0 (61.7; 87.2)
Carotid plaque, n (%)	128 (69.9)
Carotid plaque number, n (IQR)	1.00 (0.00; 2.00)
Maximal carotid artery stenosis, % (IQR)	25.0 (0.00; 33.8)
cTPA, mm^2^, Me (IQR)	22.5 (12.5; 40.5)
cPS, mm, Me (IQR)	3.00 (1.61; 4.06)
Lower extremity arteries plaque, n (%)	99 (54.1)
Lower extremity arteries plaque number, n (IQR)	1.00 (0.00; 2.00)
Total plaque number, n (IQR)	2.00 (1.00; 4.00)
Maximal lower extremity artery stenosis, % (IQR)	30.0 (0.00; 35.0)
Polyvascular atherosclerosis, n (%)	82 (44.8)
ABS	
0	39 (21.3)
1	39 (21.3)
2	48 (26.2)
3	30 (16.4)
4	27 (14.8)

Comments: BMI—body mass index; HTN—hypertension; T2DM—type 2 diabetes mellitus; CAD—coronary artery disease; RAAS—renin-angiotensin-aldosterone system; TC—total cholesterol; LDL-c—low density lipoprotein cholesterol; HDL-c—high density lipoprotein cholesterol; non-HDL-c—non-high-density lipoprotein cholesterol; HbA1c—glycated hemoglobin; eGFR—estimated glomerular filtration rate; cTPA—carotid total plaque area; cPS—carotid plaque score; ABS—atherosclerosis burden score.

**Table 2:** Serum biomarker findings.

Characteristics	Patients (n = 183)
hsCRP, mg/L, Me (IQR)	1.82 (0.90; 2.75)
Pentraxin 3, pg/mL, Me (IQR)	8.58 (3.88; 13.5)
TGFβ, pg/mL, Me (IQR)	17.4 (6.77; 36.7)
GM-CSF, pg/mL, Me (IQR)	24.5 (9.40; 54.8)
IL1β, pg/mL, Me (IQR)	1.35 (0.85; 1.93)
IL2, pg/mL, Me (IQR)	1.05 (0.55; 2.10)
IL4, pg/mL, Me (IQR)	0.00 (0.00; 22.8)
IL5, pg/mL, Me (IQR)	0.99 (0.11; 2.86)
IL6, pg/mL, Me (IQR)	0.57 (0.00; 22.8)
IL7, pg/mL, Me (IQR)	43.6 (18.9; 55.9)
IL8, pg/mL, Me (IQR)	5.09 (0.85; 10.5)
IL10, pg/mL, Me (IQR)	9.90 (4.68; 16.6)
IL12p70, pg/mL, Me (IQR)	2.79 (1.54; 5.77)
IL13, pg/mL, Me (IQR)	0.00 (0.00; 5.99)
IFNγ, pg/mL, Me (IQR)	17.8 (12.0; 23.7)
TNFα, pg/mL, Me (IQR)	7.09 (5.12; 10.4)

Comments: hsCRP—high sensitivity C-reactive protein; IL—interleukin; TGFβ—transforming growth factor-beta; GM-CSF—granulocyte macrophage colony-stimulating factor; IFNγ—interferon gamma; TNFα—tumor necrosis factor-alpha.

**Table 3:** Results of flow cytometry.

Characteristics	Patients (n = 183)	
	Absolute Number, Cells/μL	Relative Number, %
CD3^+^ cells	1406.5 (1205.0; 1686.0)	75.7 (69.1; 80.7)
CD19^+^ cells	214.0 (167.5; 307.0)	11.8 (9.52; 14.8)
CD3^−^CD56^+^ cells	164.0 (97.0; 261.0)	8.24 (5.43; 12.1)
CD3^+^CD4^+^ cells	893.0 (728.0; 1107.0)	48.5 (41.8; 54.8)
CD3^+^CD8^+^ cells	479.0 (348.5; 655.5)	23.8 (19.7; 29.4)
CD3^+^CD25^+^ cells	47.5 (32.0; 79.5)	2.54 (1.49; 3.92)
CD4^+^CD25^+^ cells	44.0 (27.2; 81.0)	2.22 (1.45; 4.05)
CD8^+^CD25^+^ cells	3.50 (0.00; 51.0)	0.25 (0.02; 2.16)
CD3^+^HLA-DR^+^ cells	36.0 (20.0; 62.0)	1.90 (1.10; 3.12)
CD4^+^HLA-DR^+^ cells	32.5 (17.5; 58.5)	1.75 (0.93; 2.88)
CD8^+^HLA-DR^+^ cells	20.5 (5.00; 50.0)	0.96 (0.23; 2.61)
CD4^+^CD25^+^CD127^−^ cells	115.0 (86)	6.00 (5.00; 7.70)
CD3^+^CD11b^+^ cells	92.0 (42.0; 149.0)	4.20 (2.30; 7.50)
CD4^+^CD11b^+^ cells	44.0 (12.2; 105.7)	2.10 (0.60; 4.85)
CD8^+^CD11b^+^ cells	86.0 (50.5; 169.7)	4.60 (2.45; 8.95)
CD14^++^CD16^−^ cells	25.0 (12.7; 46.7)	59.6 (30.7; 79.6)
CD14^++^CD16^+^ cells	25.0 (13.7; 68.7)	5.39 (2.52; 13.3)
CD14^+^CD16^++^ cells	25.0 (12.7; 46.7)	5.89 (3.41; 9.64)
CD14^++^CD16^−^TLR2^+^ cells	35.5 (12.0; 92.0)	7.73 (2.73; 28.5)
CD14^++^CD16^+^ TLR2^+^ cells	306.0 (220.0; 407.0)	71.2 (47.5; 93.8)
CD14^+^CD16^++^ TLR2^+^ cells	233.5 (92.0; 318.0)	51.9 (21.6; 91.2)
CD14^++^CD16^−^TLR4^+^ cells	216.0 (101.0; 345.0)	59.6 (23.8; 85.2)
CD14^++^CD16^+^ TLR4^+^ cells	403.5 (305.0; 493.0)	95.2 (81.7; 98.6)
CD14^+^CD16^++^ TLR4^+^ cells	374.5 (294.0; 483.0)	92.3 (77.5; 97.1)

## Data Availability

The data presented in this study are available on request from the corresponding author.

## Supplementary Materials

The following supporting information can be downloaded at: https://www.mdpi.com/article/10.3390/ijms241512205/s1, Figure S1: Gating strategy of flow cytometry. Sequential gating strategy for the identification of lymphocytes subpopulations. Figure S2: Gating strategy of flow cytometry. Sequential gating strategy for the identification of CD25^+^ T cells subpopulations. Figure S3: Gating strategy of flow cytometry. Sequential gating strategy for the identification of HLA-DR^+^ T cells subpopulations. Figure S4: Gating strategy of flow cytometry. Sequential gating strategy for the identification of T-regulatory lymphocytes subpopulations. Figure S5: Gating strategy of flow cytometry. Sequential gating strategy for the identification of monocytes subpopulations.
