# Supplementary material for: Innate and Adaptive Immunity-Related Markers as Predictors of the Short-Term Progression of Subclinical Atherosclerosis in Middle-Aged Patients
Source: Int J Mol Sci. 2023 Jul 30;24(15):12205. doi: 10.3390/ijms241512205 (PMC10419170; doi:10.3390/ijms241512205)
Supplement: Supplementary file 1 [file ijms-24-12205-s001.zip › ijms-2514630-supplementary.pdf]

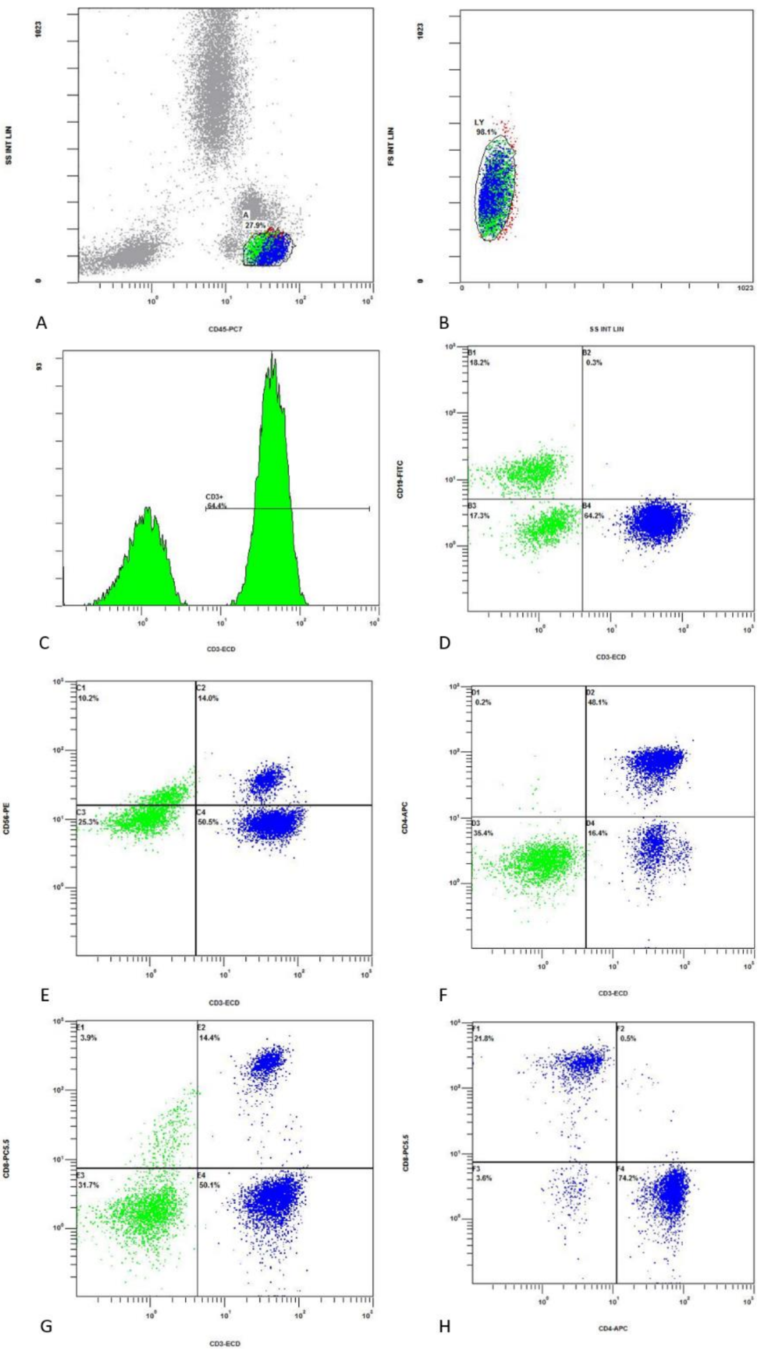

Figure S1. Gating strategy of flow cytometry. Sequential gating strategy for the identification of lymphocytes subpopulations: CD45^{+} cell identification (A), setting a logical limitation for area A based on Forward Scatter and Side Scatter (B), CD3 receptor expression histogram on white blood cells (C), identification of CD45^{+}CD3^{+}CD19^{-} corresponding to T-cells and CD45^{+}CD3^{-}CD19^{+} corresponding to B-cells phenotypes (D), identification of CD45^{+}CD3^{-}CD56^{+} phenotypes corresponding to NK-cells; CD45^{+}CD3^{+}CD56^{-} corresponding to NKT-cells (E), identification of CD45^{+}CD3^{+}CD4^{+} phenotypes corresponding to T-helper cells; CD45^{+}CD3^{+}CD8^{+} corresponding to T-cytotoxic lymphocytes; CD45^{+}CD3^{+}CD4^{+}CD8^{+} corresponding to a heterogeneous population of double positive T-cells (F-H).

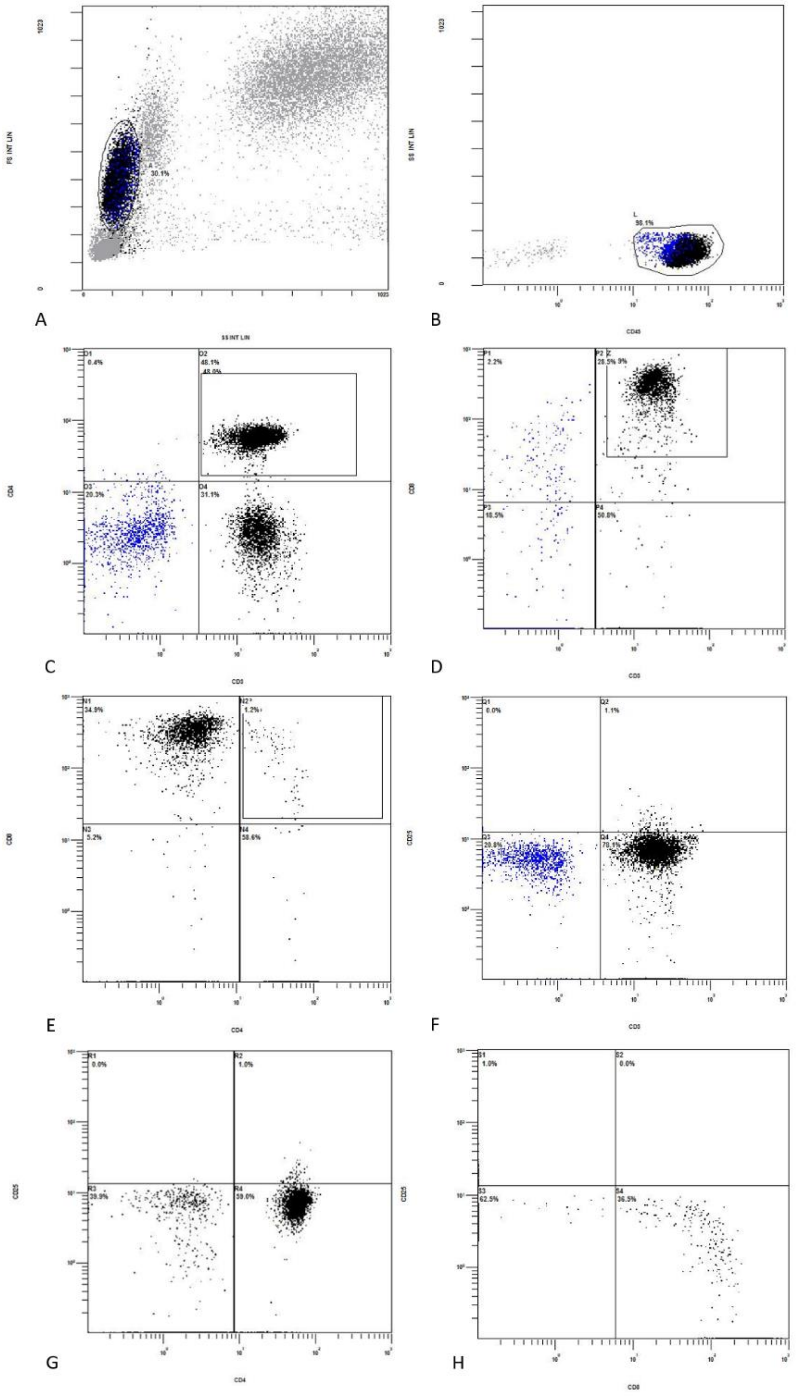

Figure S2. Gating strategy of flow cytometry. Sequential gating strategy for the identification of CD25$^+$ T cells subpopulations: setting a logical limitation for area A based on Forward Scatter and Side Scatter with lymphocyte population isolation (A), setting a logical limitation for area A with identification of CD45$^+$ cells (B), identification of CD45$^+$CD3$^+$CD4$^+$ phenotypes corresponding to T-helper cells; CD45$^+$CD3$^+$CD8$^+$ corresponding to T-cytotoxic lymphocytes; CD45$^+$CD3$^+$CD4$^+$CD8$^+$ corresponding to a heterogeneous population of double positive T-cells (C-E), identification of CD25-expressing T-cell phenotypes (including T-helper and T-cytotoxic lymphocytes) based on CD3, CD4, CD8 and CD25 expression (F-H).

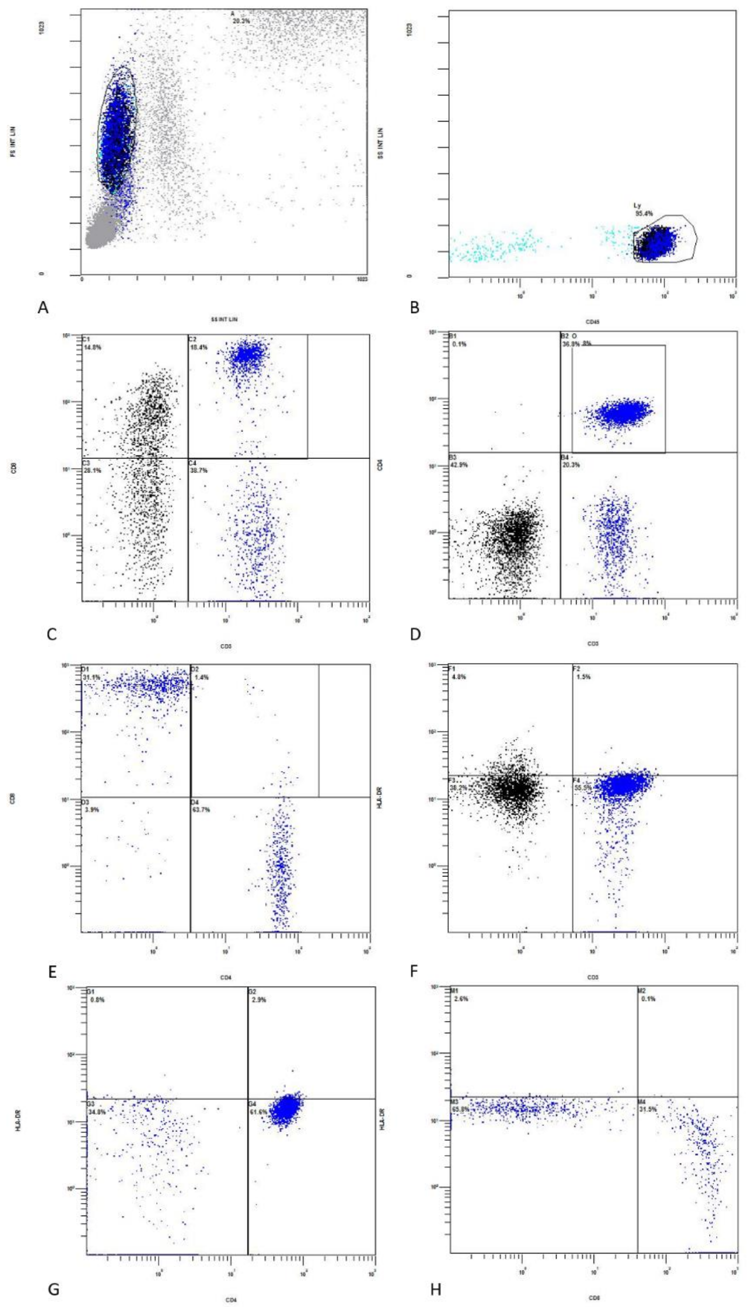

Figure S3. Gating strategy of flow cytometry. Sequential gating strategy for the identification of HLA-DR^{+} T cells subpopulations: setting a logical limitation for area A based on Forward Scatter and Side Scatter with lymphocyte population isolation (A), setting a logical limitation for area A with identification of CD45^{+} cells (B), identification of CD45^{+}CD3^{+}CD4^{+} phenotypes corresponding to T-helper cells; CD45^{+}CD3^{+}CD8^{+} corresponding to T-cytotoxic lymphocytes; CD45^{+}CD3^{+}CD4^{+}CD8^{+} corresponding to a heterogeneous population of double positive T-cells (C-E), identification of HLA-DR-expressing T-cell phenotypes (including T-helper and T-cytotoxic lymphocytes) based on CD3, CD4, CD8 and HLA-DR expression (F-H).

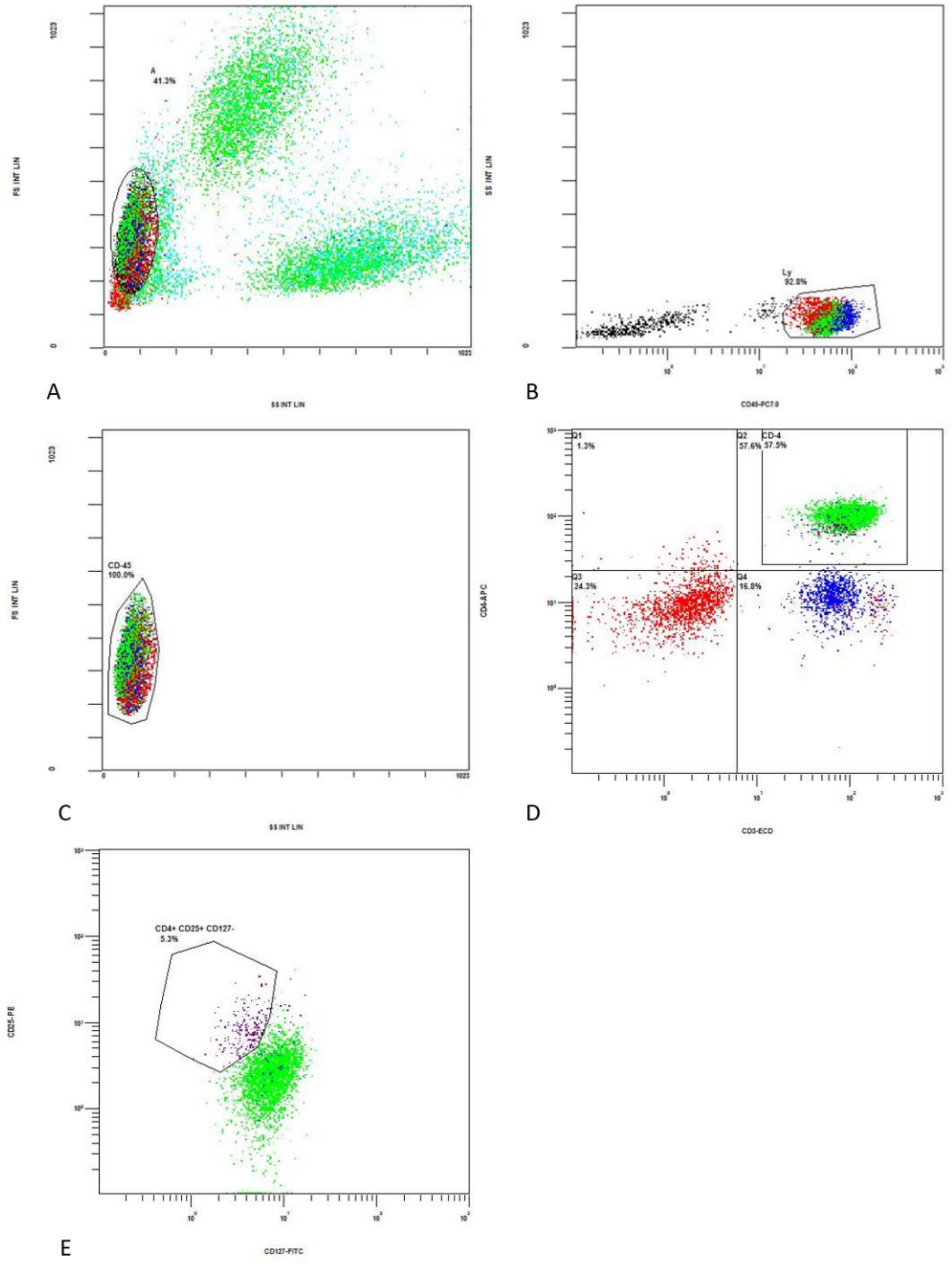

Figure S4. Gating strategy of flow cytometry. Sequential gating strategy for the identification of T-regulatory lymphocytes subpopulations: setting a logical limitation for area A based on Forward Scatter and Side Scatter with lymphocyte population isolation (A), setting a logical limitation for area A with identification of $CD45^{+}$ cells (B), setting a second logical limitation for the lymphocytic region based on Forward Scatter and Side Scatter (C), identification of $CD45^{+}CD3^{+}CD4^{+}$ phenotypes corresponding to T-helper cells (D), identification of $CD45^{+}CD3^{+}CD4^{+}CD25^{+}CD127^{-}$ phenotypes corresponding to T-regulatory cells (E).

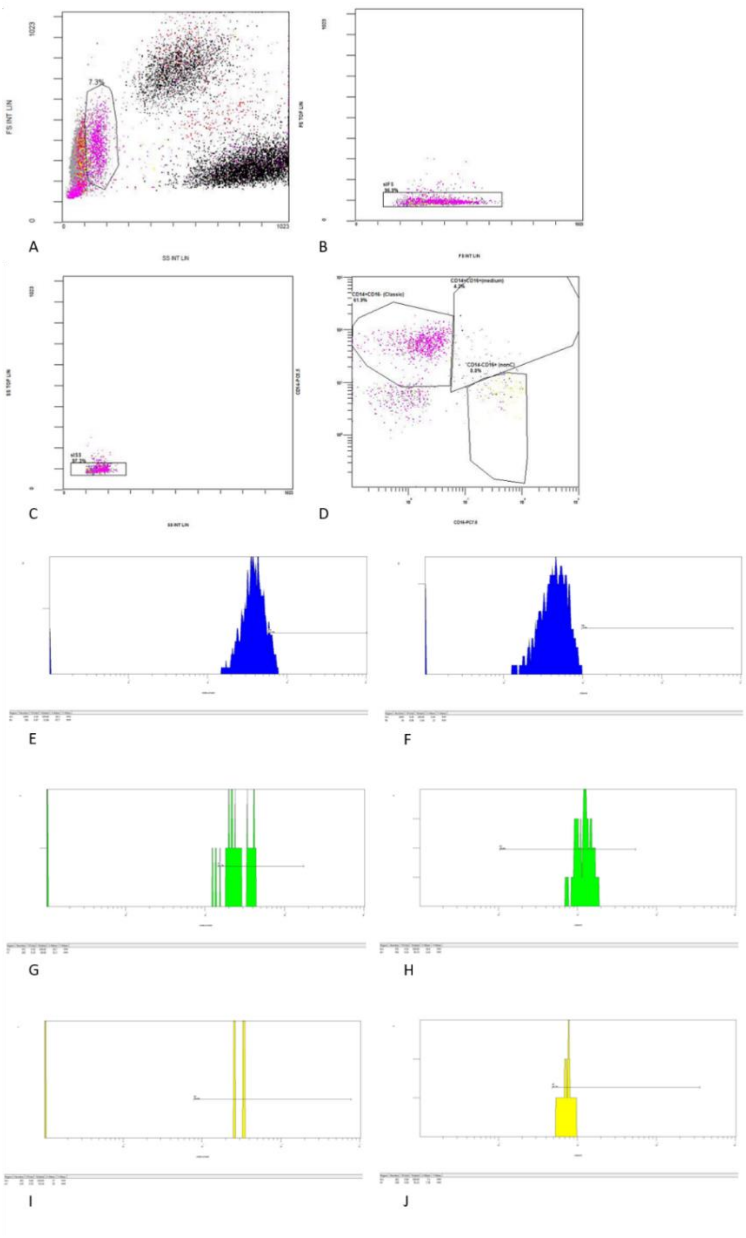

Figure S5. Gating strategy of flow cytometry. Sequential gating strategy for the identification of monocytes subpopulations: setting a logical limitation for area A based on Forward Scatter and Side Scatter with monocyte population isolation (A), gating for single cells in a FS TOF vs FS INT plot (B), gating for single cells in a SS TOF vs SS INT plot (C), identification of $CD14^{+}CD16^{-}$ phenotypes corresponding to classical monocytes, $CD14^{+}CD16^{+}$ phenotypes corresponding to intermediate monocytes, $CD14^{-}CD16^{+}$ phenotypes corresponding to non-classical monocytes (D), identification of TLR2 and TLR4 expression levels on classical monocytes, as well as the number of TLR2- and TLR4-positive classical monocytes (E-F), identification of TLR2 and TLR4 expression levels on intermediate monocytes, as well as the number of TLR2- and TLR4-positive intermediate monocytes (G-H), identification of TLR2 and TLR4 expression levels on non-classical monocytes, as well as the number of TLR2- and TLR4-positive non-classical monocytes (I-J).
